# Nano-TiO_2_ reinforced CoCr matrix wear resistant composites and high-temperature tribological behaviors under unlubricated condition

**DOI:** 10.1038/s41598-020-63918-4

**Published:** 2020-04-22

**Authors:** Gongjun Cui, Yanping Liu, Sai Li, Huiqiang Liu, Guijun Gao, Ziming Kou

**Affiliations:** 1 College of Mechanical and Vehicle Engineering, Taiyuan University of Technology, Taiyuan, 030024 PR China; 2 National-Local Joint Engineering Laboratory for Mine Fluid Control, Taiyuan, 030024 PR China

**Keywords:** Engineering, Materials science

## Abstract

The CoCrMo matrix composites with nano-TiO_2_ particle (2 wt.%, 4 wt.% and 6 wt.%) were fabricated by using a powder metallurgy technique (P/M), and the nano-TiO_2_ content was optimized in matrix. The microstructures, mechanical and high-temperature tribological properties of the synthesized composites were systematically studied. Friction and wear behaviors were studied by using a disk-on-ball tribo-tester sliding against Si_3_N_4_ ceramic ball from room temperature (23 ^o^C) to 1000 ^o^C in air. TiO_2_ obviously strengthened the hardness and high-temperature wear resistance of composites. It was attributed to the high load-carrying capacity of matrix, *in-situ* formed high-temperature solid lubricants and stable oxides film on the wear tracks. 4 wt.% TiO_2_ was the critical threshold at which there was a transition of tribological properties over a broad temperature range. The composite containing 4 wt.% nano-TiO_2_ exhibited the most reasonable high-temperature friction coefficient and wear rate at all testing temperatures. At different testing temperatures, the composites showed different wear mechanisms.

## Introduction

High-temperature wear of mechanical parts is always an inevitable problem in engines due to the absence of lubricating oil^1–3^. With the increasing power of engines, there is a strong need for the materials with the good tribological properties over a broad temperature range. It is undoubted that the materials with excellent tribological properties can reduce energy consumption^4^. Some material engineers had prepared different coatings on the surfaces of mechanical parts so as to reduce the wear^5,6^. These coatings also showed the excellent wear resistance at elevated temperatures^7–9^. Recently, the nickel and ceramic matrix self-lubricating composites were investigated due to their superior oxidation resistance and high-temperature strength at elevated temperatures^10–12^. Cobalt matrix alloys have higher mechanical properties and wear resistance than those of nickel matrix alloys at elevated temperatures^4,7^. And therefore, it is necessary to further strengthen the wear resistance of cobalt matrix materials at elevated temperatures in order to prolong the working life of mechanical parts. Additionally, Erdemir adopted a crystal-chemical model (ionic potential-*Φ*) to predict the lubricating effect of metal oxides^13^. The oxides with high ionic potential were easily sheared during sliding, and exhibited the lubricating effect at elevated temperatures^14^. The ionic potentials of TiO_2_ is 5.8, which can show lubricating effect under some special conditions. Meanwhile, TiO_2_ has a low thermal expansion coefficient and high thermal shock resistance at elevated temperatures. Hence, TiO_2_ particle is a potential reinforcement for Co matrix high-temperature wear resistant materials.

The investigations of high-temperature tribological properties of cobalt alloys had been reported, though these researchers only studied the tribological mechanisms^4,15,16^. At elevated temperatures, the alloys do not have the lubricating effect. Wear resistance of materials only depends on the metal oxides on the surface of wear tracks at elevated temperatures, and oxidation also causes the degradation of materials^4,17^. Whereas the mechanical properties of materials directly influence the wear rate at low temperatures. Thus, the friction coefficients and wear rates of Co matrix materials are relatively high. Li and Radu^17–19^ adopted the yttrium (Y) to reinforce the wear resistance of different Stellite alloys at 600 °C. Y element formed compounds which strengthened the mechanical properties of alloys. The Y_2_O_3_ improved the oxides scale on the contact surfaces. The addition of solid lubricants is the common way to strengthen the friction and wear properties of materials at elevated temperature, such as fluorides, oxides, soft metals, etc.^10,20,21^. Heterogeneous solid lubricants destroy the continuity of matrix of materials, resulting in the decrease in wear resistance and mechanical properties of materials. To solve this problem, many scientists try to find some means to improve the wettability between solid lubricants and metal matrix. Ceramic particles were widely chosen as strengthening phase to improve the high-temperature tribological properties of materials. Botto *et al*.^22^ studied the wear resistance of Co matrix coatings with Al_2_O_3_ from RT to 1000 ^o^C. Micro-Al_2_O_3_ increased the modulus of elasticity that influenced the wear rates of coatings. The coating showed the highest friction coefficient when Al_2_O_3_ content reached up to 30 wt.%. Prasad *et al*.^23^ prepared CoMoCrSi-Cr_3_C_2_ coating and studied the tribological properties from 200 to 600 °C. Cr_3_C_2_ obviously reinforced the wear resistance of CoMoCrSi matrix coating in comparison with that of substrate. The obtained results indicated that ceramic particles were effective as strengthening phase for wear resistant coatings. Jayabharathy *et al*.^24^ chose TiO_2_ particle to strengthen the tribologcial properties of AZ91 magnesium matrix composite at room temperature. They found that TiO_2_ improved the wear resistance of alloy with a slight increase in friction coefficient. Nageswaran *et al*.^25^ found that micro-TiO_2_ could decrease the friction coefficients of Cu matrix composites at dry-sliding condition, and Cu-9%TiO_2_-1% Gr composite had better wear resistance. Nowadays, the wear resistant cobalt matrix composite reinforced by TiO_2_ is rarely done at elevated temperatures.

According to the above discussion, in this study, the CoCrMo alloy was selected as the matrix of composites, and nano-TiO_2_ particles acted as the reinforcement of composites. Meanwhile, the TiO_2_ content in matrix was optimized. CoCrMo matrix composites were prepared by P/M. The high-temperature friction and wear behaviors of composites were conducted on a ball-on-disk tribotester sliding against Si_3_N_4_ ceramic ball from room temperature (23 °C) to 1000 °C in air. The wear and friction mechanisms were discussed.

## Experimental Procedure

### Specimens

The starting materials in this study were commercial Co powder (64 μm, 99.7% purity), Cr powder (53 μm, 99.8% purity) and Mo powder (70 μm, 99.7% purity). The size of nano-TiO_2_ (Rutile) was about 25 nm. The composition of composites was listed in Table 1 (denoted as CT0, CT2, CT4 and CT6). The powders were ball-milled by a high energy mill machine according to the composition of composites. The ratio of powders to ball was 1:10. The milling time was 6 hours and the rotational speed was 200 rpm. Four composites were sintered by using a powder metallurgy technique (P/M). Firstly, the mixed powders were put into a graphite die (inner diameter: 30 mm), and then the graphite die was set in a vacuum hot-pressing furnace and heated to 1100 ^o^C when the pressure of furnace was evacuated to 10^−2^ Pa. The heating rate was about 10 ^o^C/min. A pressure of 35 MPa was held at 1100 ^o^C for 40 minutes. Specimens were naturally cooled down.

**Table 1:** Composition of sintered Co matrix composites (mass%).

Specimens	Co	Cr	Mo	Nono-TiO_2_
CT0	72	18	10	0
CT2	70	18	10	2
CT4	68	18	10	4
CT6	66	18	10	6

### Tribological tests

**Figure 1:**
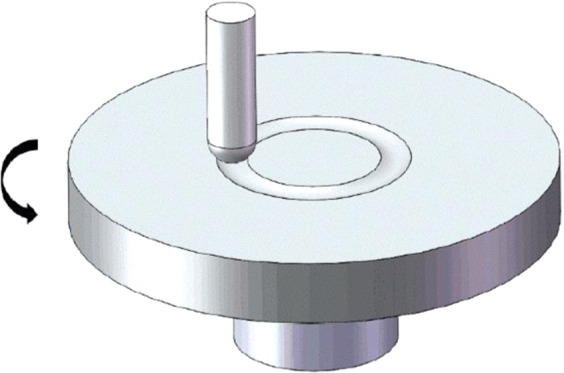
Configuration of high-temperature tribo-tester.

The high-temperature tribological properties of specimens were evaluated by using a ball-on-disk tribotester from 23 ^o^C to 1000 ^o^C in air (see Fig. 1). The ball was the commercial Si_3_N_4_ ceramic ball (diameter: 6 mm) which was fixed. The specimens were cut into disks with a dimension of 20 mm × 20 mm × 4 mm. Before each test, the surface of disks was polished to a surface roughness of 0.27 μm (Ra) with 1500 grit emery paper under cooling water condition, and cleaned by ultrasonic cleaner. The tribological experiment simulated the working conditions of high-temperature bearing in engine. The tests were conducted at 0.20 m/s, and the applied load was 10 N^26^. The turning radius was 5 mm with testing time 20 minutes. The testing temperatures were 23 ^o^C (room temperature), 200 ^o^C, 400 ^o^C, 600 ^o^C, 800 ^o^C and 1000 ^o^C. Each testing point was done three times in order to ensure the accuracy of data.

Microstructures and wear morphologies of the composites were analyzed by using scanning electron microscopy (SEM) and energy dispersive spectroscopy (EDS). The phases in matrix were determined by using X-ray Diffraction (XRD). The Vickers hardness of specimens was tested by using a Vickers hardness tester (Load: 300 g, Dwell time: 10 s). The hardness was tested ten times, and average value was reported in this study. The three-point bending strength of specimens was tested. The dimension of specimens was 3 mm × 3 mm × 20 mm, and the speed of crosshead was 3.3 × 10^−4^s^−1^. The density of composites was calculated according to the Archimedes’ method. The cross profile of specimens were tested by using a contact surface profiler. The specific wear rate of specimens was determined by the following formula:

1 $${\boldsymbol{W}}=\frac{{\boldsymbol{V}}}{{\boldsymbol{N}}\bullet {\boldsymbol{S}}},$$

where *W* is the specific wear rate of specimens (mm^3^/N.m). *V* is the wear volume (mm^3^), *S* is the sliding distance (m) and *N* is the normal load (N).

## Results and Discussion

### Microstructures and physical properties of specimens

**Figure 2:**
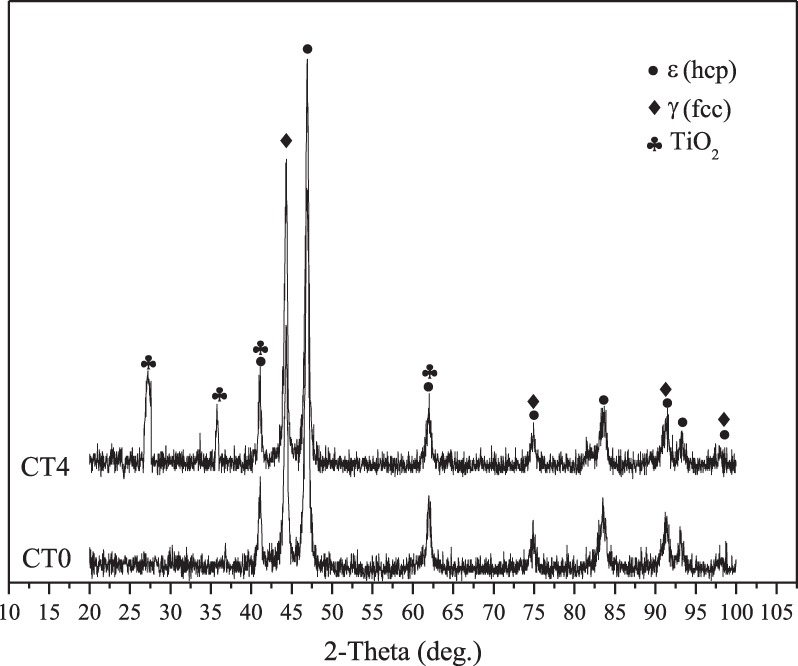
XRD patterns of CT0 and CT4.

Figure 2 gives the XRD patterns of CT0 and CT4. Because the composition of CT2 and CT6 is similar to that of CT4, the XRD pattern of CT4 is given in the figure. Cr and Mo have high solid solubility in the crystal of Co according to their phase diagram^27^. Cr and Mo atoms enter into the lattice of Co due to the solid state reaction at elevated temperatures, and form the ε (hcp) phase and γ (fcc) phase^15,18^. And therefore, the metal matrix of composites consists of two allotropes: low-temperature stable ε (hcp), and high-temperature stable γ (fcc). As can be seen from figure, the diffraction peaks of TiO_2_ are detected from the matrix. It indicates that the nano-TiO_2_ does not react with other metal elements at high temperature and the main phases of specimen CT4 are ε (hcp), γ (fcc) and nano-TiO_2_.

**Figure 3:**
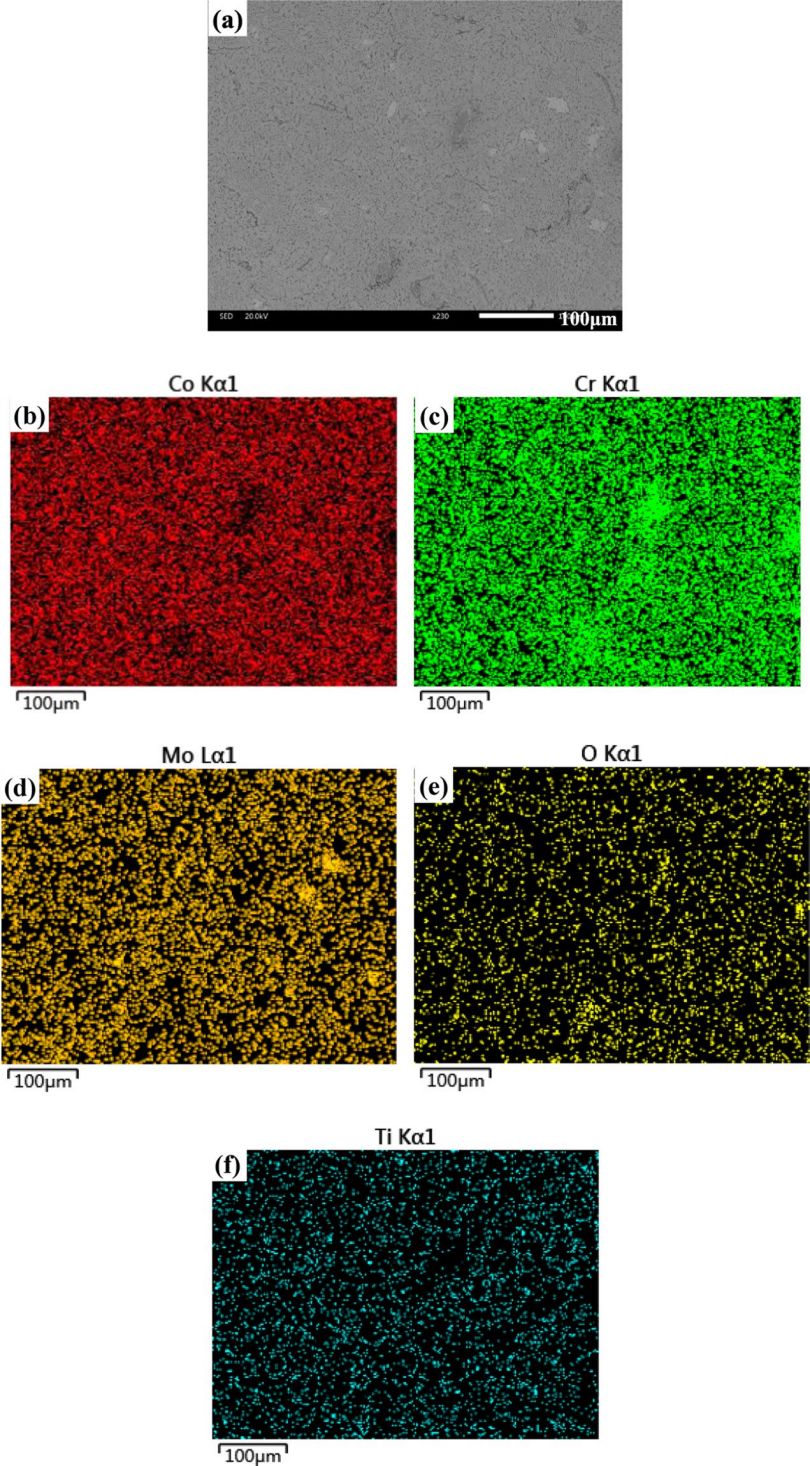
Microstructure of CT4 and the corresponding elemental maps.

The microstructure and elemental maps of CT4 are given in Fig. 3. Obvious hole and crack are not noted on the surface of specimens, and the composite shows a good compactness which would ensure the excellent mechanical and tribological properties (see Fig. 3a). The distribution of Co, Cr and Mo is basically uniform throughout the matrix according to the EDS analysis (see Fig. 3b–d). Co, Cr and Mo fully react at high temperature. It indicates that the light grey area is the ε (hcp) and γ (fcc). The distribution of Ti and O is dispersive in the matrix (see Fig. 3e,f). It means that the dark grey area is the TiO_2_-rich phase in matrix.

**Figure 4:**
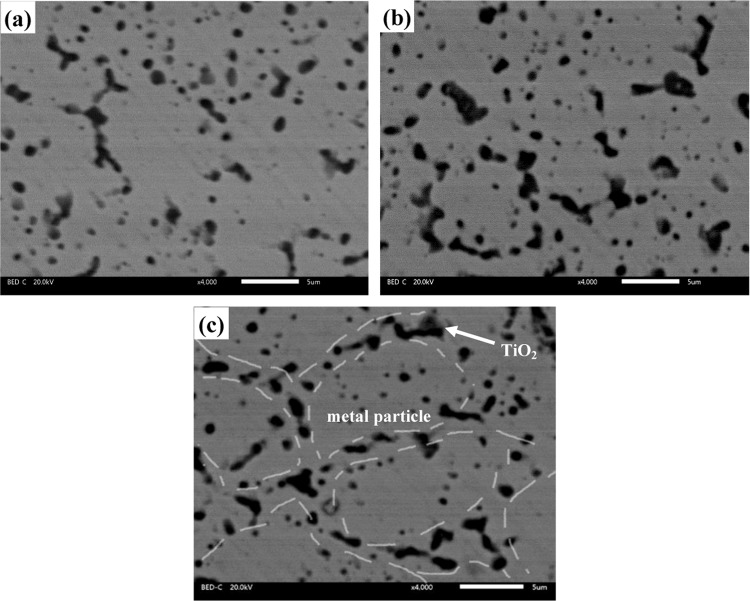
Distribution of nano-TiO_2_ at the interfaces of metal particles: (**a**) CT2, (**b**) CT4 and (**c**) CT6.

Table 2 illustrates the mechanical properties, porosity and density of different composites. The nano-TiO_2_ distributes along the interface of metal particles. These fine metal oxide can hinder the motion of dislocation. Meanwhile, the second-phase particle also prevents the crack initiation and growth. Thus, the TiO_2_ particle shows the dispersion strengthening effect on Co-Cr-Mo matrix. And therefore, the nano-TiO_2_ improves the Vickers hardness of specimens. The hardness increases with the increasing TiO_2_ content. When the nano-TiO_2_ content increases, the more TiO_2_ particles aggregate at the interfaces of metal particles (see Fig. 4), which stops the metal atom from diffusing during solidification in order to decrease the bonding strengthen of metal particles. The TiO_2_ particles deteriorate the continuity of matrix. And therefore, the bending strength of composites decreases with the increase of TiO_2_ content. This phenomenon was also obtained in Fe_3_Al-TiC composites^28^. The density of composites shows a downward trend on the ground of the low density of TiO_2_. Due to poor wettability of metal matrix and ceramic particles, tiny voids unavoidable accumulate between the two, and it happens with higher mass percent of TiO_2_. Although the porosity of specimens increases, the composites show the good compactness (see Fig. 3).

**Table 2:** Mechanical and physical properties of sintered composites.

Specimens	Hardness	Bending strength (MPa)	Density (g/mm^3^)	Porosity
CT0	383 ± 7	1634 ± 15	8.57	0.92%
CT2	573 ± 9	1427 ± 20	8.38	1.01%
CT4	589 ± 10	1372 ± 13	8.20	1.13%
CT6	609 ± 10	1323 ± 15	8.02	1.22%

### Tribological properties

**Figure 5:**
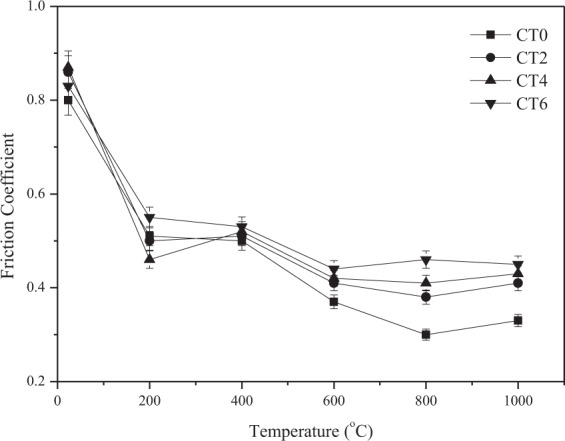
Friction coefficients of sintered composites with temperature at 10 N and 0.20 m/s.

The vibrations of friction coefficients of different composites with temperature at 10 N and 0.20 m/s sliding against Si_3_N_4_ ball are given in Fig. 5. The specimens show a similar trend. The friction coefficients decrease as the testing temperatures rise. Otherwise, the friction coefficients are high for all composites at room temperature. The specimens with nano-TiO_2_ particles have slightly high friction coefficients compared to those of specimen CT0. Meanwhile, the friction coefficients of CT2, CT4 and CT6 increase with the increasing TiO_2_ content under experimental conditions. Generally speaking, the CT6 shows the highest friction coefficients at all testing temperatures. Additionally, the TiO_2_-free CT0 shows the lowest friction coefficients during sliding. The mechanism will be discussed below.

**Figure 6:**
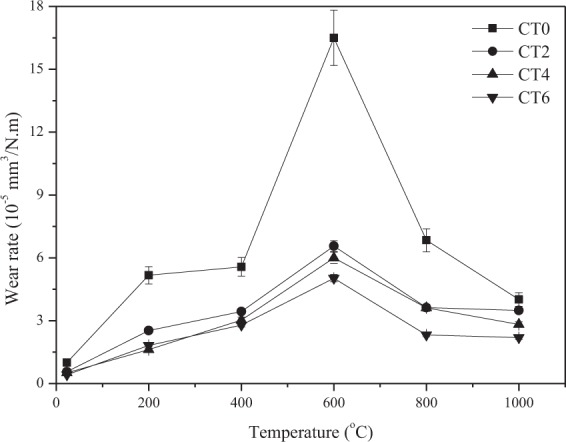
Wear rates of sintered composites with temperature at 10 N and 0.20 m/s.

Figure 6 shows the specific wear rates of four composites with temperature at 0.20 m/s and 10 N sliding against Si_3_N_4_ ball. The wear rates of all composites increase when temperature reaches up to 600 ^o^C, and then decrease with the increase of testing temperature. CT2, CT4 and CT6 show the higher wear resistance than that of CT0 from 23 ^o^C to 1000 ^o^C. The wear rate of CT0 is about 1.65 × 10^−4^ mm^3^/N.m at 600 ^o^C, those of CT2, CT4 and CT6 is about 10^−5^ mm^3^/N.m. Additionally, a distinct decrease in wear rate of specimens containing TiO_2_ particle is due to the increasing TiO_2_ content in matrix. Overall, the CT6 keeps the lowest wear rates at the testing conditions. According to the obtained results, the prepared target composites show much higher wear resistance than those of Stellite 6-Y and Stellite 712-Y (about 50 × 10^−2^ mm^3^/N.m), and the working temperature of Co matrix composites with nano-TiO_2_ is 1000 ^o^C higher than those of Stellite 6-Y and Stellite 712-Y^18,19^.

**Figure 7:**
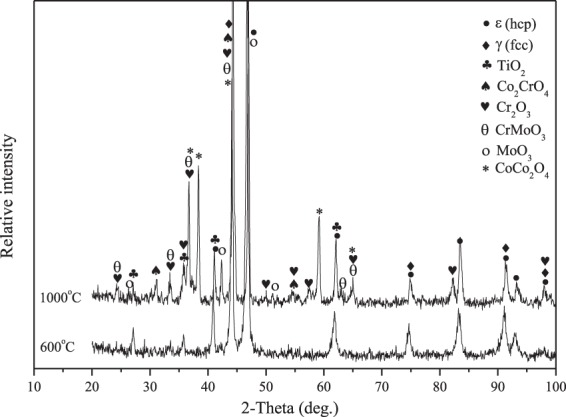
XRD patterns of the wear tracks of CT4 at 600 ^o^C and 1000 ^o^C.

**Figure 8:**
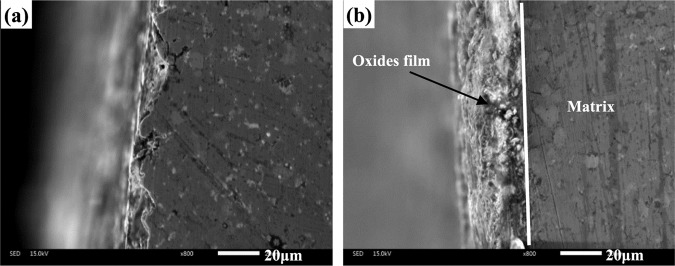
SEM images of cross section for CT4 at different temperatures: (**a**) 600 ^o^C and (**b**) 1000 ^o^C.

There is no doubt that the nano-TiO_2_ particle plays an important part in influencing the wear and friction behaviors from room temperature to 1000 ^o^C. The wear rates of materials are dependent on the hardness of materials^29,30^. The nano-TiO_2_ has a dispersion strengthening effect on the hardness of Co matrix composites as second-phase particle, resulting in the increase of hardness of materials. Thus, the wear resistance of the composites increases significantly with increasing of TiO_2_ content. The hard TiO_2_ phase can support the part of external load in order to protect material from removing during sliding. The specimen CT6 has the highest hardness so that CT6 shows the lowest wear rates at elevated temperatures. Other researchers also reported the similar research results^23,31^. For another, with the further increase of temperature, the ε (hcp) phase transfers to γ (fcc) phase in order that the ductility increases above 400 ^o^C^2^, that is, the hardness of materials decreases. Meanwhile, at low temperatures, the amount of metal oxides is small on the worn surfaces in order that the process of oxidation-oxides removal-oxidation occurs (see Fig. 7). These factors destroy the wear resistance of composites at 600 ^o^C (see Fig. 6). With increasing TiO_2_ content, more TiO_2_ particles expose on the wear tracks during sliding. These particles can scratch the contact surfaces of tribo-couples in order to increase the sliding resistance. Meanwhile, the spalling TiO_2_ particles would contribute to the friction coefficient of composites as third-body. Herein, the friction coefficients of composites increase as the TiO_2_ content reaches up to 6 wt.% over a wide temperature range. The high-temperature oxidation is a positive factor for the tribology of materials^4,11,32,33^. The metal oxides and other compounds can form oxides film on the wear tracks, and the high coverage of oxides film leads to the low wear rates and friction coefficients at elevated temperatures. Co, Cr and Mo elements are oxidized, and formed Co_2_CrO_4_, CrMoO_3_, CoCo_2_O_4_ and metal oxides at elevated temperatures (see Fig. 7). The *in-situ* formed salt compounds and metal oxides can provide lubricating effect for the composites as high-temperature solid lubricants^9,34^. At low temperatures, these compounds do not form obvious oxides film on the wear tracks (see Fig. 8a). Whereas the specimen can form a stable oxides film on the wear track at high temperatures (see Fig. 8b). Therefore, the friction coefficient and wear rate decrease when the testing temperature further increases. By comprehensive consideration, the CT4 with 4 wt.% TiO_2_ shows the optimum friction coefficients and wear rates under the given conditions.

### Worn surfaces analysis

**Figure 9:**
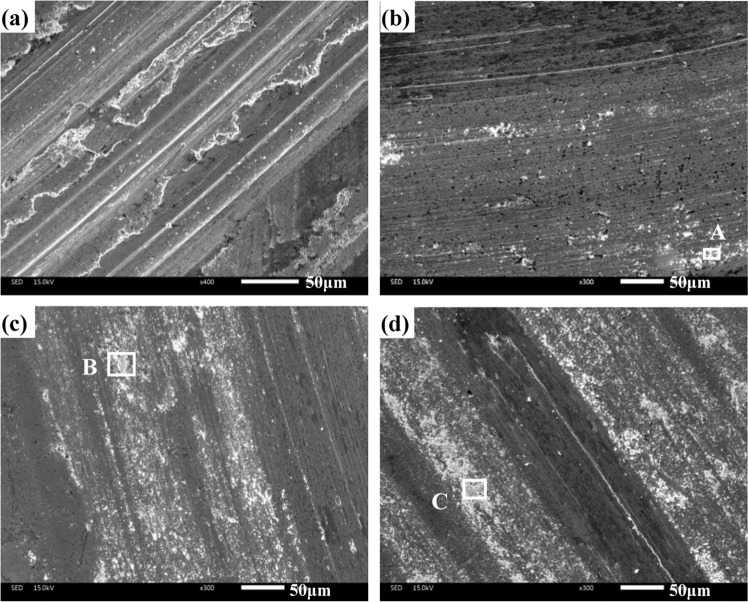
Worn surfaces of different specimens at room temperature: (**a**) CT0, (**b**) CT2, (**c**) CT4 and (**d**) CT6.

**Figure 10:**
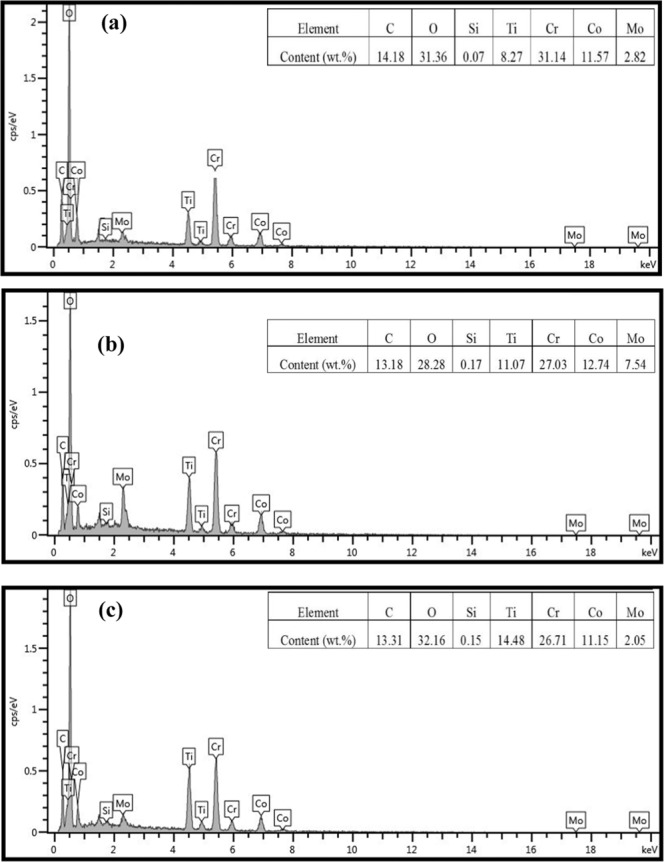
EDS analysis of wear debris of areas A, B and C in Fig. 9.

**Figure 11:**
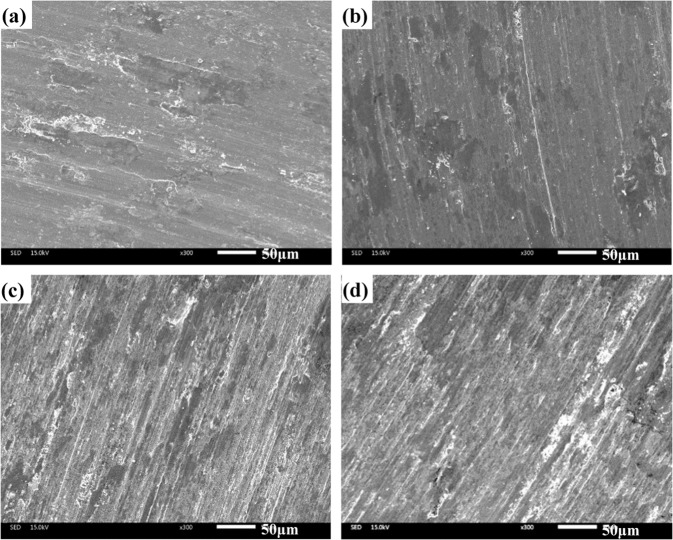
Worn surfaces of four composites at 600 ^o^C: (**a**) CT0, (**b**) CT2, (**c**) CT4 and (**d**) CT6.

Figure 9 illustrates the SEM images of obtained composites at room temperature. The ploughing and plastic deformation are noted on the wear tracks of specimen CT0 (see Fig. 9a). The worn surfaces of CT2, CT4 and CT6 become smoother than that of specimen CT0, and the slight ploughing and wear debris are obvious on the wear tracks (see Fig. 9b–d). It implies the transition in the wear mechanism of all composites. The TiO_2_ particles act as the obstacles for ductility of Co matrix alloys, which restrict the movement of dislocation in matrix. And therefore, the property resistance to plastic deformation of composites with TiO_2_ is improved. Moreover, TiO_2_ particles peel off from the worn surfaces and become the wear debris. The TiO_2_-rich wear debris ploughs the contact surfaces in order to cause the grooves (see Fig. 10). The cutting resistance can increase the friction coefficients of composites at low temperature. The wear mechanism of specimens is abrasive wear at room temperature. Figure 11 gives the worn morphologies of composites at 600 ^o^C. The grooves and plastic deformation are noted on the wear tracks of CT0 (see Fig. 11a). During the friction process, the wear debris is ground by the tribo-couples and becomes the patch that adheres to the worn surfaces. CT2, CT4 and CT6 show the different morphologies in comparison with that of CT0 (see Fig. 11b–d). The characteristics of grooves become more and more obvious when TiO_2_ content increases in matrix. When the testing temperature rises and reaches the phase inversion temperature of ε(hcp)→γ(fcc), the hardness of matrix decreases and plasticity increases. The TiO_2_ particles easily groove the ploughing on the worn surfaces. Meanwhile, the amount of oxides is low so that the oxides film is not obvious on the wear tracks due to the low temperature (see Fig. 8a). It corresponds to the higher wear rates of composites at 600 ^o^C. The wear mechanism of specimens is characterized by the abrasive wear and slight oxidation wear at 600 ^o^C.

**Figure 12:**
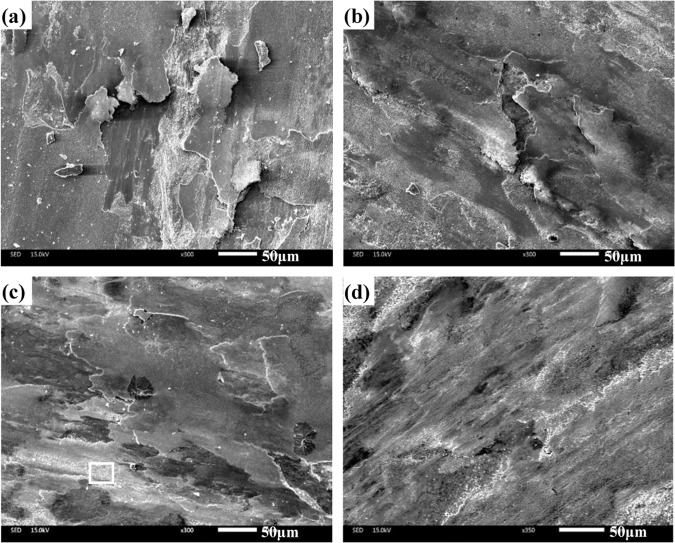
SEM images of worn surfaces of different composites at 1000 ^o^C: (**a**) CT0, (**b**) CT2, (**c**) CT4 and (**d**) CT6.

**Figure 13:**
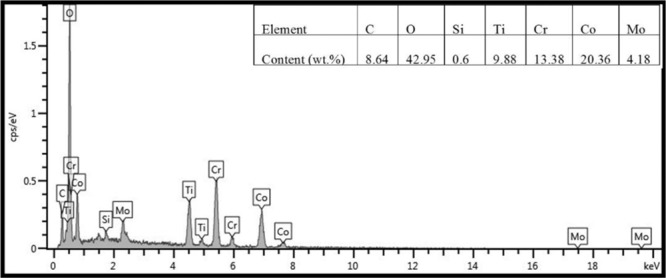
EDS analysis of oxides film on the worn surface in Fig. 12c.

The worn morphologies of four composites at 1000 ^o^C are presented in Fig. 12. At 1000 ^o^C, the smeared oxides film is noted on the worn surfaces for all specimens, which consists of complex compounds and metal oxides^4,17,18^ (see Fig. 7). In this case, the process of oxidation-oxides removal-oxidation is inhibited. The oxides film segregates the tribo-couples during sliding in order to change the wear model, which leads to the low wear rate and friction coefficient of composites at elevated temperatures^34^. It is clear that the oxides film of CT2, CT4 and CT6 becomes more and more intact in comparison with that of CT0. The external load can cause the plastic deformation of the substrate due to the low hardness of CT0 in order that the oxides film is destroyed on the contact surfaces. After losing the oxides film, the fresh materials must be exposed on the sliding surfaces, resulting in an increase of wear rates at 1000 ^o^C. Nevertheless, the addition of TiO_2_ decreases the ductility and improves the hardness of materials^28^. Specimens containing TiO_2_ can support the stable oxides film at 1000 ^o^C because of the high hardness. But the oxides film also includes nano-TiO_2_ particles, and the actual content of TiO_2_ is higher than nominal content (see Fig. 13), thereby increasing the friction coefficients of specimens. This indicates that the main wear mechanism is the oxidation wear at elevated temperatures.

**Figure 14:**
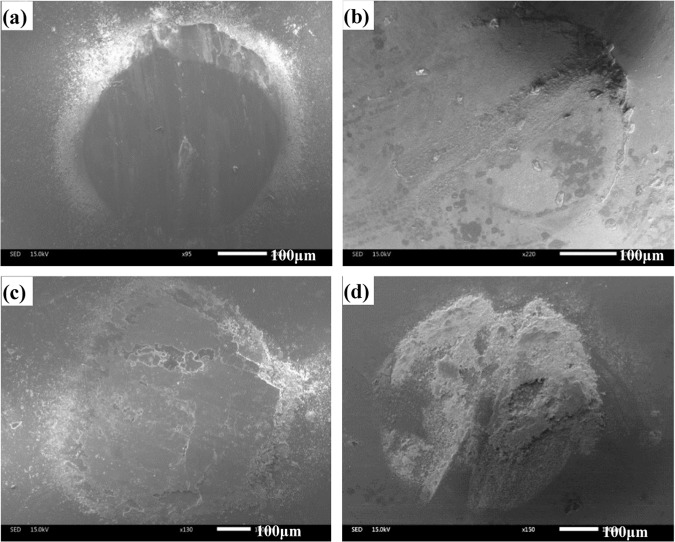
SEM images of worn surfaces of Si_3_N_4_ balls sliding against CT4 at: (**a**) RT, (**b**) 600 °C, (**c**) 800 °C and (**d**) 1000 ^o^C.

Figure 14 gives the worn surfaces of Si_3_N_4_ ceramic ball sliding against CT4 at different testing temperatures. The ceramic ball offers the relatively smooth worn surfaces at low temperatures (room temperature and 600 ^o^C). Meanwhile, the transferred layer is rare on the contact surfaces at room temperature and 600 ^o^C (see Fig. 14a,b). However, at 800 ^o^C and 1000 ^o^C, the transferred layer is obvious on the wear scars of ceramic ball (see Fig. 14c,d). The transferred layer can reduce the contact area of tribo-couples during sliding process, resulting in an improvement in the friction and wear of Co matrix composites at elevated temperatures^35,36^.

## Conclusions

1. The phases of CoCr matrix composites consisted of low-temperature stable ε (hcp), high-temperature stable γ (fcc) and nano-TiO_2_. The distribution of each ingredient was uniform in matrix. The hardness of sintered composites increased with the increasing of TiO_2_ content.
2. The friction coefficients of composites increased when the TiO_2_ content increased because the nano-TiO_2_ particles scratched the worn surfaces of tribo-couples. However, wear rates showed the opposite trend. It attributed to the high hardness and stable oxides film. Co_2_CrO_4_, CrMoO_3_, CoCo_2_O_4_ and metal oxides formed on the worn surfaces. These compounds constituted the lubricating film (oxides film). The specimens containing nano-TiO_2_ could bear and form a stable oxides film on the wear tracks because of the high hardness. The wear rates of specimens with nano-TiO_2_ were lower than those of nano-TiO_2_-free specimen at testing temperatures.
3. The specimen with 4 wt% TiO_2_ had the most reasonable high-temperature friction and wear properties from room temperature to 1000 ^o^C due to the synergistic effect of high mechanical properties, *in-situ* formed solid lubricants and stable oxides film.
4. When the testing temperature was below 600 ^o^C, the wear mechanism of composites was the abrasive wear. At 1000 ^o^C, the main wear mechanism was the oxidation wear.
